# Documentation of Mandated Birth Certificate Data Elements Across US Birth Settings

**DOI:** 10.1001/jamanetworkopen.2025.11615

**Published:** 2025-05-20

**Authors:** Amos Grünebaum, Frank A. Chervenak

**Affiliations:** 1 Donald and Barbara Zucker School of Medicine at Hofstra/Northwell, Hempstead, New York

## Abstract

This cross-sectional study evaluates documentation rates of mandated birth certificate elements, including 5-minute Apgar scores, to assess compliance with federal requirements.

## Introduction

The 5-minute Apgar score assesses 5 critical indicators of neonatal well-being: heart rate, respiratory effort, muscle tone, reflex irritability, and skin color. Federal law mandates national collection and publication of births and other vital statistics data.^1^ This cross-sectional analysis evaluated documentation rates of select birth certificate elements, including the 5-minute Apgar score, across birth settings to assess compliance with federal requirements and identify potential documentation gaps.

## Methods

Using CDC National Center for Health Statistics birth certificate data^2^ (2016-2023), we assessed documentation rates for 4 validated birth certificate elements^3^: 5-minute Apgar score, birth weight, delivery method, and gestational age. We restricted the analysis to midwife-attended births (certified nurse midwives or others [including licensed midwives and traditional midwives where applicable]) across hospitals, freestanding birth centers, and intended home births to ensure consistency in documentation practices and standardized birth attendance across settings. Deliveries that were unplanned or not attended by a midwife were excluded. Elements with explicitly stated values were considered documented; those coded as “unknown or not stated” were classified as undocumented. We analyzed documentation rates and temporal trends using χ^2^ tests with relative risk ratios and linear regression. Model diagnostics confirmed that the data met all assumptions required for linear regression analysis. *P* < .05 was considered significant. This study followed the STROBE reporting guideline and qualifies as exempt under the Common Rule for the use of deidentified data.

## Results

The study population included 3 253 364 births (2 859 643 hospital, 156 531 birth center, and 237 190 home births) (Table). Compared with hospital births, Apgar score documentation rates were significantly lower in intended home births (RR, 0.97; 95% CI, 0.97-0.97; *P* < .001) and birthing centers (RR, 0.98; 95% CI, 0.98-0.98; *P* < .001). Documentation rates remained consistently high across settings for gestational age, birth weight, and delivery mode. Longitudinal analysis demonstrated stable documentation rates in hospitals (slope, 0.009 [95% CI, −0.003 to 0.021] percentage points/y; *P* = .13) but not in birth centers (slope, −0.007 [95% CI, −0.063 to 0.049] percentage points/y; *P* = .78) or home births, which showed a significant decline from 97.40% in 2016 to 96.29% in 2023 (slope, −0.143 [95% CI, −0.230 to −0.056] percentage points/y; *P* = .008), representing a 42.7% increase in undocumented cases from the 2016 baseline of 2.60% (Figure).

**Table.** Birth Certificate Documentation Rates by Birth Setting, 2016-2023

Data element	Documented, No. (%)		
	Hospital (n = 2 859 643)	Birthing center (n = 156 531)	Home birth (n = 237 190)
5-min Apgar	2 855 919 (99.87)	153 638 (98.15)	229 902 (96.93)
Gestational age	2 858 852 (99.97)	156 531 (100)	236 603 (99.75)
Birth weight	2 858 776 (99.97)	156 446 (99.95)	236 264 (99.61)
Delivery mode	2 858 928 (99.97)	156 505 (99.98)	236 419 (99.67)

**Figure.**
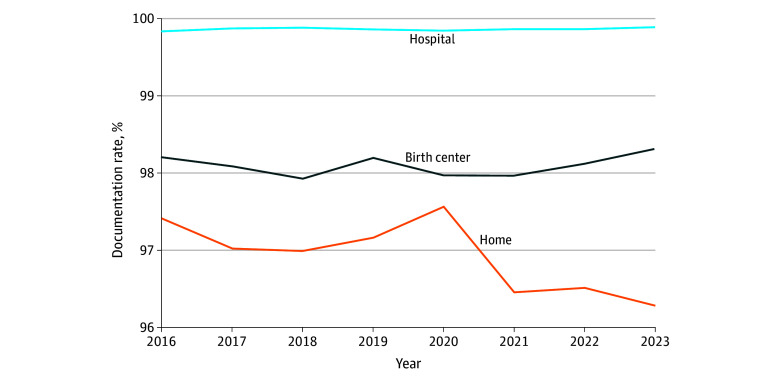
5-Minute Apgar Score Documentation by Birth Setting, 2016-2023 The graph shows documentation rates across hospital (n = 2 859 643), birth center (n = 156 531), and home birth (n = 237 190) settings. Hospital rates remained stable at approximately 99.87% (slope, 0.009 [95% CI, −0.003 to 0.021] percentage points/y; *P* = .13), while home birth documentation declined significantly from 97.40% to 96.29% (slope, −0.143 [95% CI, −0.230 to −0.056] percentage points/y; *P* = .008).

## Discussion

Our analysis revealed marked systematic variations across birth settings in the documentation of 5-minute Apgar scores. While most required elements showed documentation rates above 99.6% in all settings, 5-minute Apgar scores were documented less frequently in out-of-hospital settings. This disparity was most pronounced in intended home births, where already suboptimal compliance rates further declined during the study period, while documentation rates remained consistently high in hospitals and birthing centers. A low 5-minute Apgar score (<7), particularly a score of 0 to 3, represents a critical indicator of neonatal mortality risk and is associated with substantially increased risk of cerebral palsy.^4^ Given extensive epidemiological evidence demonstrating significantly elevated neonatal morbidity and mortality rates among out-of-hospital births,^5,6^ systematic documentation of this vital assessment is imperative.

Several factors may have influenced the declining documentation rates in home births, including a lack of standardized oversight, varying documentation practices, medicolegal concerns, and philosophical differences among practitioners. In some cases, low Apgar scores might have been intentionally or unintentionally omitted, leading to artificially inflated outcome statistics and a distorted representation of home birth safety. In addition, our analysis could not account for potential state-level variations in documentation practices. These issues underscore the need for standardized and transparent reporting to ensure accurate assessments of home birth outcomes. While hospitals undergo Joint Commission accreditation with regular documentation audits, out-of-hospital birth settings lack comparable standardized oversight of documentation practices. Implementation of standardized documentation training and audit protocols across birth settings could improve adherence to federal documentation requirements and enhance birth certificate data quality.

## Notes

Supplement.
Data Sharing Statement
