# Supplementary material for: Documentation of Mandated Birth Certificate Data Elements Across US Birth Settings
Source: JAMA Netw Open. 2025 May 20;8(5):e2511615. doi: 10.1001/jamanetworkopen.2025.11615 (PMC12093183; doi:10.1001/jamanetworkopen.2025.11615)
Supplement: Supplement. — Data Sharing Statement [file jamanetwopen-e2511615-s001.pdf]

Data Sharing Statement

Grünebaum. Documentation of Mandated Birth Certificate Data Elements Across US Birth Settings. *JAMA Netw Open*. Published May 20, 2025.
doi:10.1001/jamanetworkopen.2025.11615

Data
Data available: Yes
Data types: Deidentified participant data
How to access data: Available online on the WONDER database
When available: With publication

Supporting Documents
Document types: Statistical/analytic code
How to access documents: Sent in
When available: With publication

Additional Information
Who can access the data: Anyone
Types of analyses: For any purpose
Mechanisms of data availability: Online
